# The Family Check-Up Online Program for Parents of Middle School Students: Protocol for a Randomized Controlled Trial

**DOI:** 10.2196/11106

**Published:** 2018-07-18

**Authors:** Brian G Danaher, John R Seeley, Elizabeth A Stormshak, Milagra S Tyler, Allison S Caruthers, Kevin J Moore, Lucia Cardenas

**Affiliations:** ^1^ Prevention Science Institute University of Oregon Eugene, OR United States

**Keywords:** family treatment, parent-child treatment, school mental health, internet intervention, eHealth intervention, prevention science, family relations, parent-child relations, school health services, mental health services, telehealth, preventive psychiatry, preventive health services

## Abstract

**Background:**

Research has established that skillful family management during adolescence protects youth from a variety of mental health and behavioral problems. Interventions associated with this research have focused on parenting skills as the mediator that links early risk factors with a profile of later behavioral risk, including problem behavior, substance use, and school failure. Fortunately, positive changes in family management skills have been linked to meaningful improvements in adolescent behavior, and these improvements have been significant across a variety of cultural groups.

**Objective:**

We describe the background, research design, and intervention components of an electronic health version of the Family Check-Up program that is targeting middle school children and is being evaluated in a randomized controlled trial for its usability, feasibility, and efficacy.

**Methods:**

We used an iterative formative research process to develop an electronic health version of the Family Check-Up program. In our ongoing randomized controlled trial, eligible families are randomly assigned to 1 of 3 conditions: Family Check-Up Online-only (n≈100), Family Check-Up Online + Coach (n≈100), and a waitlist control condition (middle school as usual; n≈100). We are conducting assessments at baseline, 3 months following randomization (posttest), and at follow-ups scheduled for 6 months and 12 months.

**Results:**

This randomized controlled trial project was funded in 2015. Participant recruitment was completed in spring 2018 and enrollment is ongoing. Follow-up assessments will be completed in 2019.

**Conclusions:**

The innovative Family Check-Up Online program has the potential to help address many of the barriers that more traditional school-based behavioral mental health implementation strategies have yet to solve, including staffing and resources to implement family-centered support within schools.

**Trial Registration:**

ClinicalTrials.gov NCT03060291; https://clinicaltrials.gov/ct2/show/NCT03060291 (Archived by WebCite at http://www.webcitation.org/70f8keeN4)

**Trial Registration:**

RR1-10.2196/11106

## Introduction

### Background

Although many young people make the transition to adolescence with only minor behavioral problems and school-related difficulties, a significant number of at-risk youth develop problem behaviors that are serious, that may last a lifetime, and that could impair later functioning. During the past decade, substance use has remained a serious public health concern, with 35% of eighth graders reporting having tried alcohol and associated increases in substance use during the adolescent years [1]. Early adolescence (ages 11-14 years) is a time of rapid biological and social transition. Interactions between parents and their child’s middle school are significantly more formalized and less frequent than in elementary school [2]. As a result, parents tend to become less involved in their child’s overall adjustment, which may subsequently lead to a variety of behavioral and social problems in high school. As a result, middle school is an ideal developmental period for family-centered prevention that targets reduction of problem behavior and substance use through teaching and supporting effective parenting skills.

Research during the past two decades has established that skillful family management, including applying positive parenting skills, setting limits, monitoring, and effectively solving problems, during adolescence protects youth from a variety of mental health and behavioral problems. Most family-centered intervention studies have focused on parenting skills as a direct target of intervention, guided by a theoretical model whereby parenting skills are the mediator that links early risk with a profile of later behavioral risk, including problem behavior, substance use, and school failure [3-6]. Fortunately, positive changes in parental family management skills have been linked to meaningful improvements in adolescent behavior across family cultures and ethnic groups [7,8]. Even among adolescents who exhibit risk, such as affiliation with deviant peers, improved family management skills by parents has been shown to decrease the growth of externalizing behavior during adolescence [9].

In this paper we describe the background, research design, and intervention components of an ongoing project funded by the US National Institute on Drug Abuse (R01DA037628) that is intended to develop and test the usability, feasibility, and efficacy of an Internet-based version of the Family Check-Up (FCU) program as a universal prevention intervention that targets middle school children. In Multimedia Appendix 1 we present the summary statement generated by peer reviewers in the US National Institute on Drug Abuse Study Section prior to our research being funded.

### Efficacy of the Family Check-Up Program

The FCU is a strengths-based, family-centered intervention that promotes family management and parent skill enhancement and addresses child and adolescent adjustment problems [10]. It has two components: (1) an ecological strengths-based self-report assessment of child behavior, parenting skills, family dynamics, and life stressors, followed by focused feedback; and (2) parent management training, which focuses on supporting positive behavior, setting healthy limits, supervision, and building relationships [11]. Depending on the particular program configuration used, the FCU family feedback session can be held at the family home, a clinic, a school, or a community center, and its delivery is typically facilitated by a counselor or coach (in school settings) or a therapist (in community mental health settings). The FCU can be delivered as both a preventive checkup and as an intensive intervention for high-risk families.

Multiple federally funded grants have examined the FCU in randomized controlled trials based in public schools that involved ethnically and socioeconomically diverse young children and middle-school–aged youths [10,12-15]. Strong effects have been found on both proximal and distal outcomes, including substance use, health behavior, and depression. The FCU delivered in middle school has been linked to long-term improvement in academic outcomes (self-regulation, grade point average, school attendance and engagement, and teacher-rated child problem behavior over time) [14,16,17] and various nonacademic outcomes (eg, depression, substance use, and high-risk sexual behavior) [17-20]. It was also related to decreased arrest rates, problem behavior, and substance use. These positive effects have been found to persist through high school and the early-adult years [5,21,22].

The FCU was found to have direct effects on putative mediators, such as youth self-regulation, and on outcomes such as deviant peer affiliation, substance use, and family conflict [17,23]. When the FCU was delivered in schools, teachers reported reduced problem behavior across the 3 years of middle school [14] and in school-related outcomes [24]. The putative mediators associated with changes in behavior across these intervention trials included parenting skill enhancement and youth self-regulation [17,23], with a particular focus on positive parenting across the life span. Improved self-regulation during the middle school years predicted reduced risk behavior during the transition to adulthood more than 10 years later [20].

### Electronic Health Interventions

Electronic health (eHealth) interventions delivered via the internet are rapidly being developed for a wide variety of target behaviors, and they have shown encouraging efficacy in controlled trials, for example, for smoking cessation [25-27], depression treatment [28-30], and obesity management [31,32].These programs can be stand-alone (fully automated), which reduces their cost of delivery while greatly increasing their reach (their public health impact), or they can include live contact with coaches or counselors in face-to-face sessions or through telephone calls [33], which increases participant adherence through accountability to a coach who is seen as trustworthy, benevolent, and having expertise. Mohr et al [33] posited a model of *supportive accountability* that describes how participant engagement and follow-through in eHealth interventions can be encouraged by the human support provided by a coach, for example, when participants receive brief calls from the coach. Using the term *coach* implies that the coach’s interaction with families need not require the skills of a highly trained clinician [34]. This level of coach support has been found to enhance the efficacy of eHealth interventions for tobacco cessation [35-37] and depression [38-40].

A number of studies have examined Web-based parent-training programs. Some programs have adopted a video teleconference approach to enable coaches or therapists to observe family interactions and guide treatment activities at parents’ homes (eg, the work of Comer and colleagues [41-43] on internet-facilitated Parent Child Interaction Therapy). Other parent-training programs include multimedia and program content designed for parents to use on their own or under the guidance of trained coaches. Examples of these studies are Incredible Years [44], a mobile phone–based version of Helping the Noncompliant Child [45], a Web-based implementation of the Strongest Years program delivered in Sweden [46], the Parenting Wisely program [47], some of the tests of the Triple-P Online program [48,49], and ezParent, a tablet-based intervention designed for a low-income, ethnic minority population of parents [50].

### Rationale for the Project

Although a variety of parent interventions in public schools have motivated positive change in parenting and reduced problem behavior [12,51,52], few children and adolescents ever receive treatment for these problems when interventions are fully disseminated, and only a very small percentage of parents participate in parenting or family interventions to address behavioral problems [53,54]. Several likely reasons could explain this problem, including inadequate funding for implementation, schools’ competing priorities, complicated logistical requirements for treatments, inadequate time for teachers and staff to be trained and to deliver the program, and parents who are difficult to recruit [55,56]. In a randomized effectiveness trial of the FCU model, we found that schools were unable to administer the FCU to families in a systematic way, although schools were generally supportive of delivering family-centered interventions from the school. The lack of trained staff and time for implementation were key factors that limited the uptake of the intervention, which was associated with improved outcomes for high-risk students, such as increased parental monitoring and decreased negative school contacts, despite poor implementation [20,57]. This research inspired our efforts to develop an eHealth intervention version of the FCU that could be administered to families with little or no staffing from schools. We used an iterative approach to development that was guided by family and school focus groups, testing of the eHealth version, and adaptations based on feedback. In the next section, we describe our development process, intervention modules, and study design.

## Methods

### Technology Development Process

We used an integrated technology architecture for the FCU Online website, its administration website, and coach portal, which involved sharing a common database. This resulted in a seamless development process that enhanced quality control and user data tracking. Program components were fully tested on a preproduction server before being moved to the live production environment.

### Program Components

**Figure 1:**
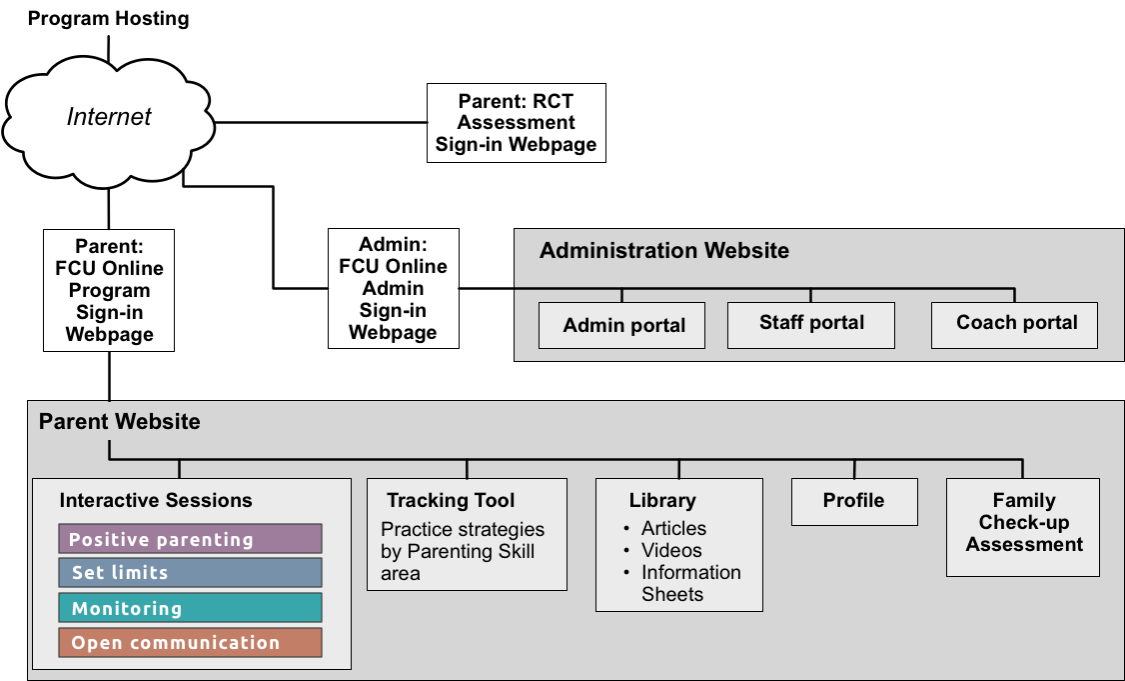
Conceptual schematic highlighting 3 Family Check-Up (FCU) Online components: the parent website, the administration website for managing administration (Admin) and staff, coaches, and guests, and the randomized controlled trial (RCT) assessment website that parents access to complete assessments.

The FCU program comprises 3 separate but complementary entities: assessment and feedback, skills sessions in the parent website, and an administration website (Figure 1).

#### Family Check-Up Program Online Assessment and Feedback

The namesake activity for the FCU is the 88-item, 23-webpage FCU assessment that participants complete as their initial step in the program. This assessment incorporates items and subscales from the Strengths and Difficulties Questionnaire [58] augmented with additional items drawn from other sources and content developed internally by the Oregon group [59]. Once participants submit their completed assessment, they receive feedback in a printout arranged according to major themes and 3 colors that convey how their child and family data compare with normative data (ie, normal, borderline, and clinical ranges). Feedback is guided by motivational interviewing principles, and it provides choices for treatment options and highlights strengths and potential areas of improvement [10]. *Green* highlights a family’s areas of strength that, when continued, will have a strong positive impact. *Yellow* signals that an area could use some attention. It does not always mean a significant problem but, if ignored, the problem behavior could escalate. *Red* indicates that an area may be a serious concern for their child or family. If no attempt is made to work on and improve serious concerns, the behavior is unlikely to improve on its own. Feedback also conveys practical changes parents can make to improve their child’s behavior and the quality of their family’s interactions.

#### Skills Sessions Website

**Figure 2:**
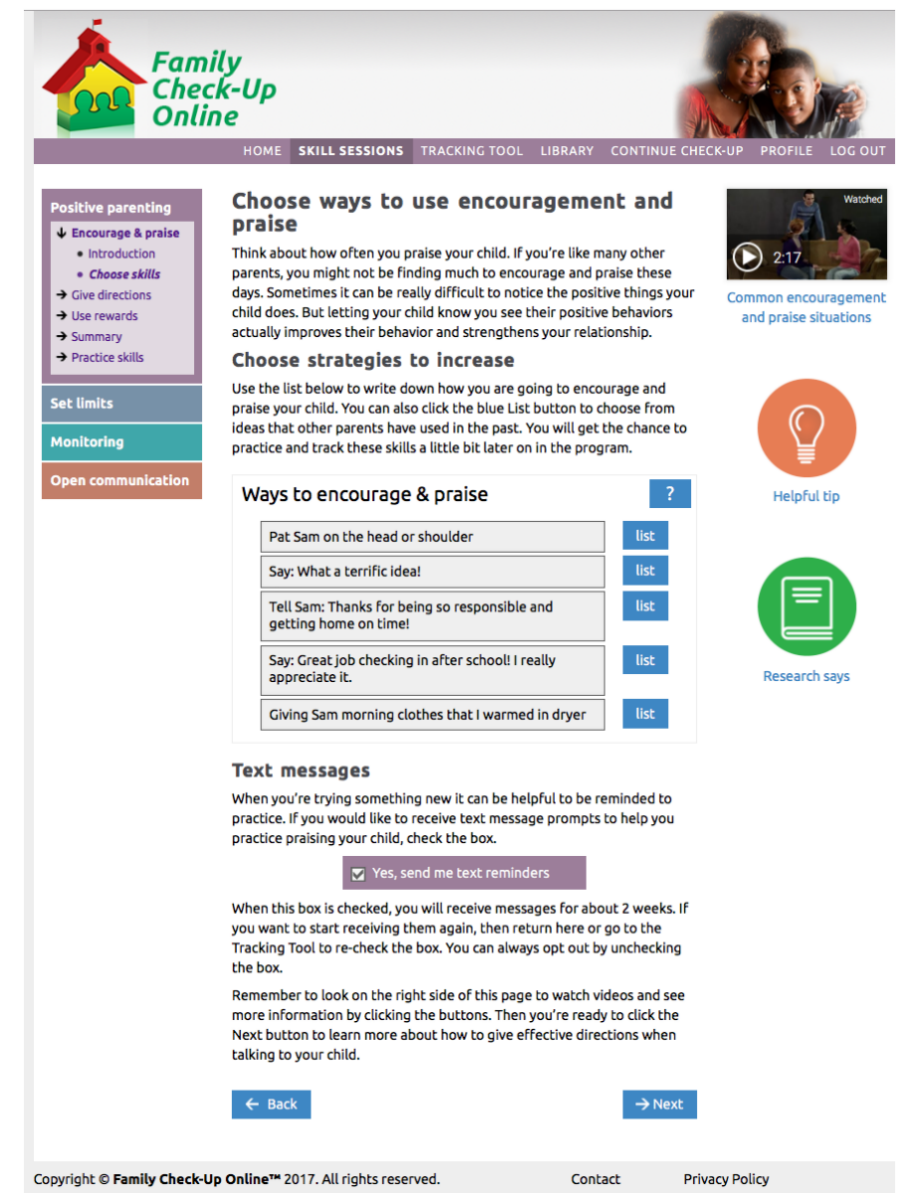
The Family Check-Up Online skills session for participants on positive parenting. Image shows top menu, left navigation, list activity for choosing skills, text messaging opt-in, video model, and additional information features (helpful tip and research says).

**Figure 3:**
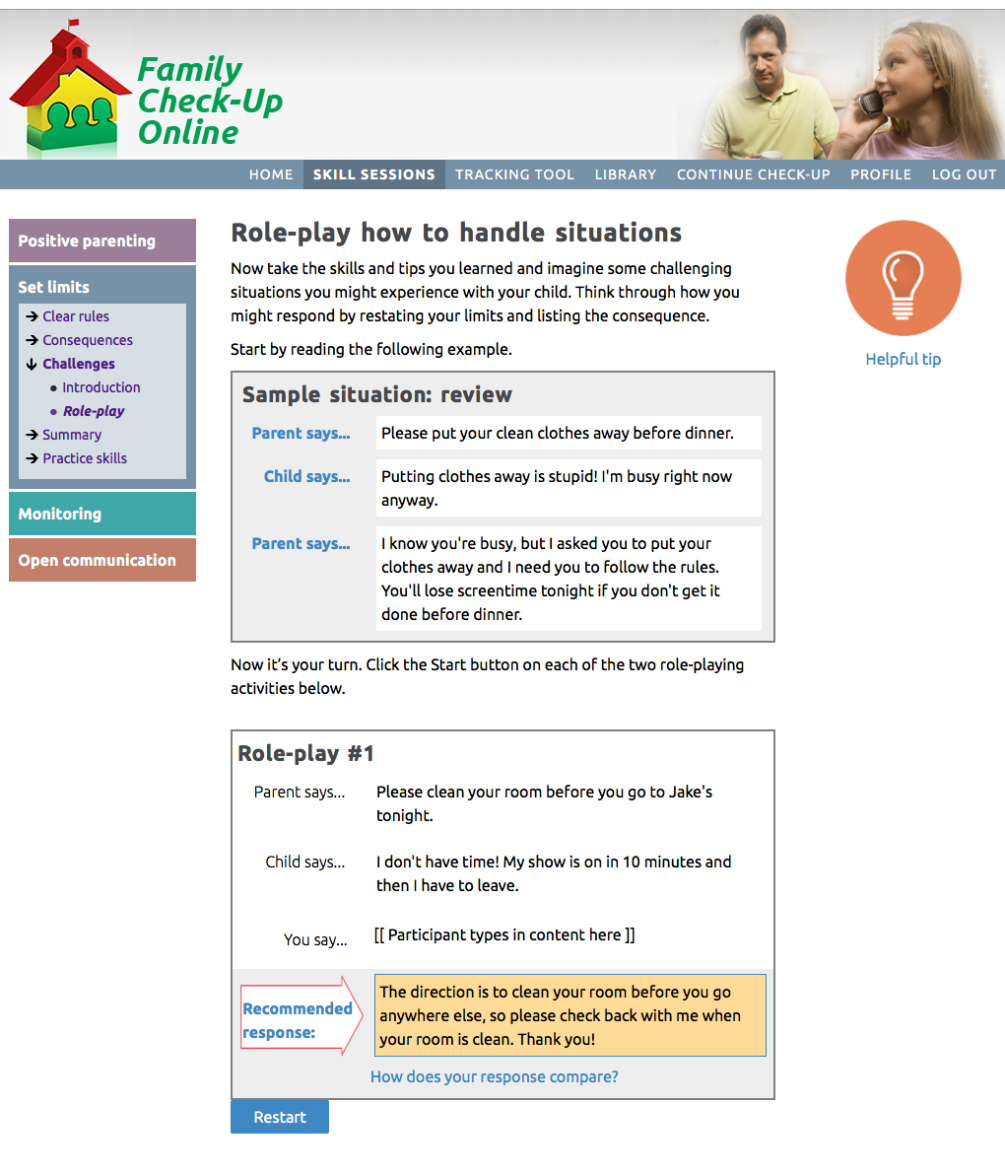
The Family Check-Up Online role-playing activity located in the participants’ set limits skills session. Image shows a role-playing activity after the participant has typed in content adjacent to the “You say” box, which triggers display of a recommended response.

**Figure 4:**
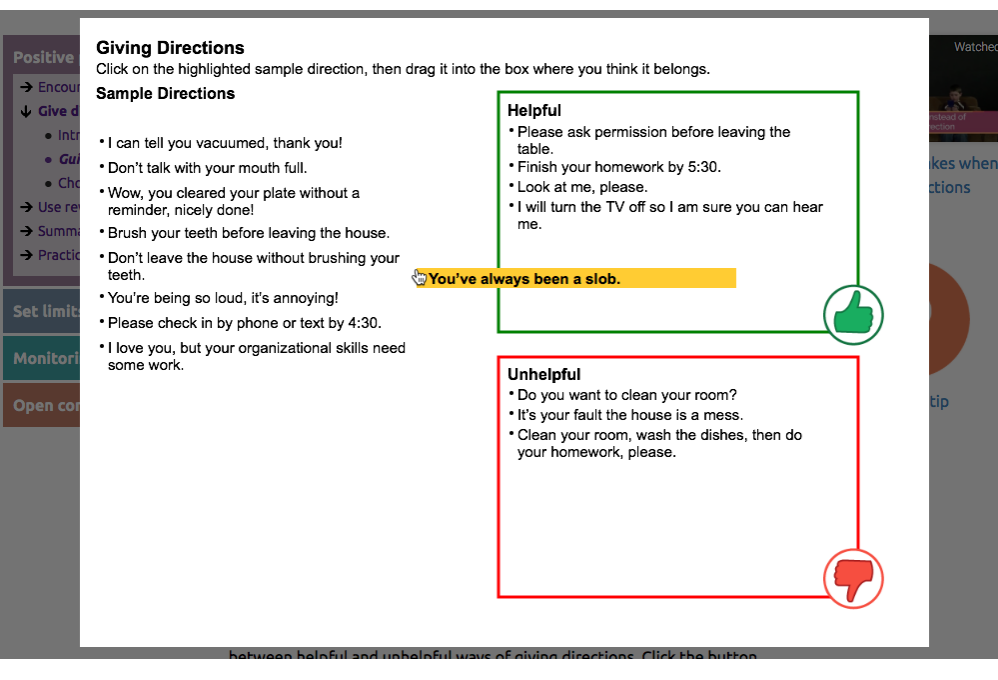
The Family Check-Up Online sorting activity located in the participants’ positive parenting skills session. Audio and written messages ask participants to drag the highlighted sample direction to a box indicating whether it is helpful or unhelpful. The program provides detailed audio feedback until all sample directions have been addressed.

Once they complete the online checkup assessment and receive related feedback, participants in the FCU Online program are able to access a set of 4 Web-based skills sessions designed to improve the ways in which they interact with their children through skills-based learning. The sessions provide the basis for personalized behavioral adjustments that can directly lead to improvement in overall family well-being. The skills sessions are the following:

- Positive parenting (Figure 2): reinforcing positive behavior through use of encouragement and praise; learning to give directions in a clear and simple way with follow-through; using rewards and incentives to reinforce desirable behavior.
- Setting limits (Figures 3 and 4): creating reasonable rules that clearly state desired behaviors and following up with predefined consequences when children do not cooperate, including consistent reinforcement of compliance.
- Monitoring: recognizing potential risks associated with increased unsupervised time that children may experience during adolescence, and improving monitoring practices to support success at home and at school.
- Open communication: using open communication and understanding that it is key to having positive family relationships; using effective parenting skills, such as listening to their children, asking questions, and problem solving.

These 4 parenting skills sessions use online engagement activities (see Table 1) that are designed to encourage the user to interact with, and be engaged with, the program. We have developed and confirmed the value of similar engagement activities in our earlier research on eHealth interventions [28-30]. Engagement activities include host videos, dyad videos that model right ways and wrong ways, animations (bear videos) that model right ways, and animated explanation of self-management and problem solving. The program also uses automated text messaging (short message service [SMS]) and emails to push or proactively send program content to users rather than relying only on the parents’ initiative to access the intervention [60,61].

**Figure 5:**
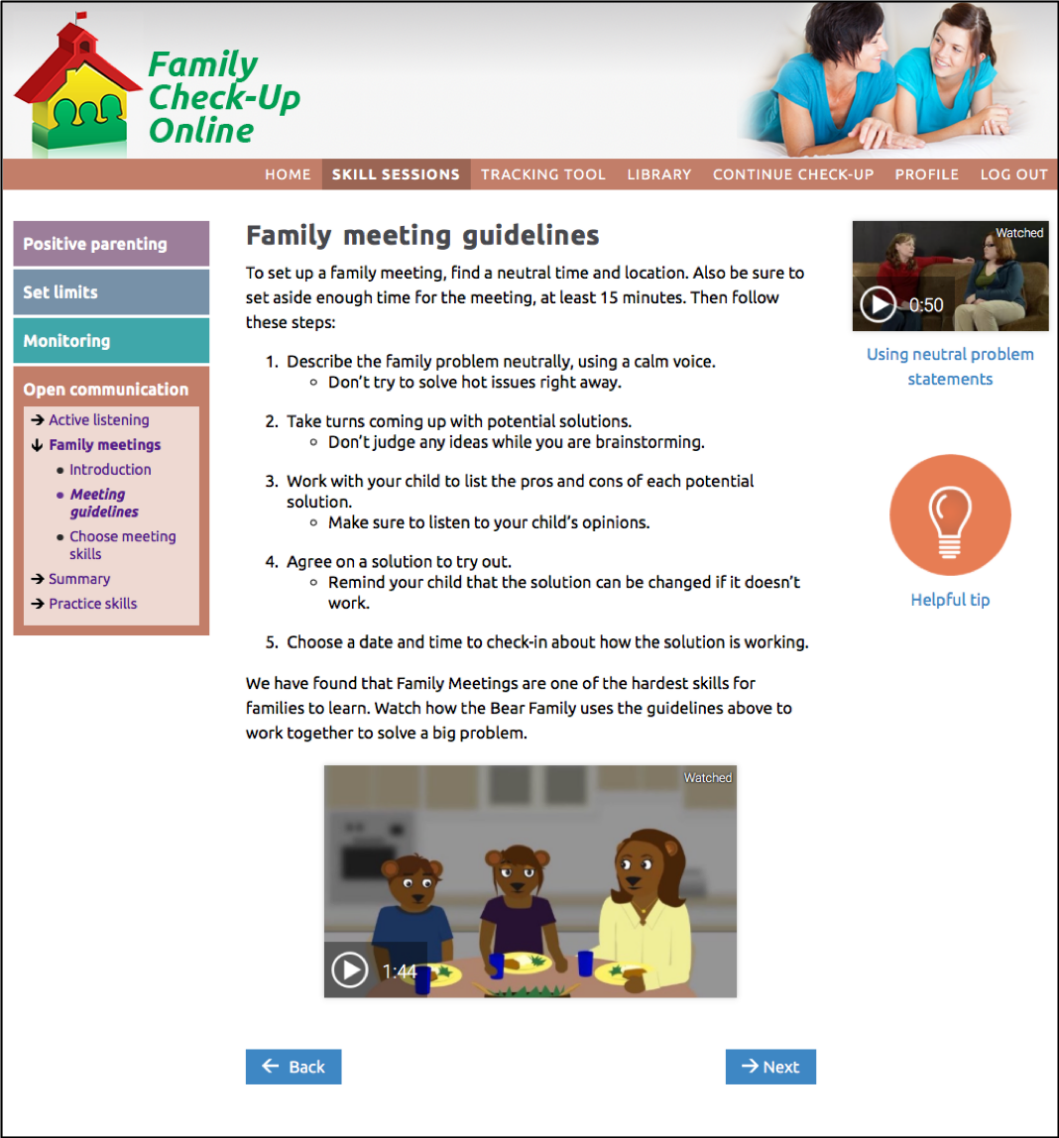
Example of both the animated bear video and the more traditional video model located on the participants’ Family Check-Up Online open communication skills session.

**Figure 6:**
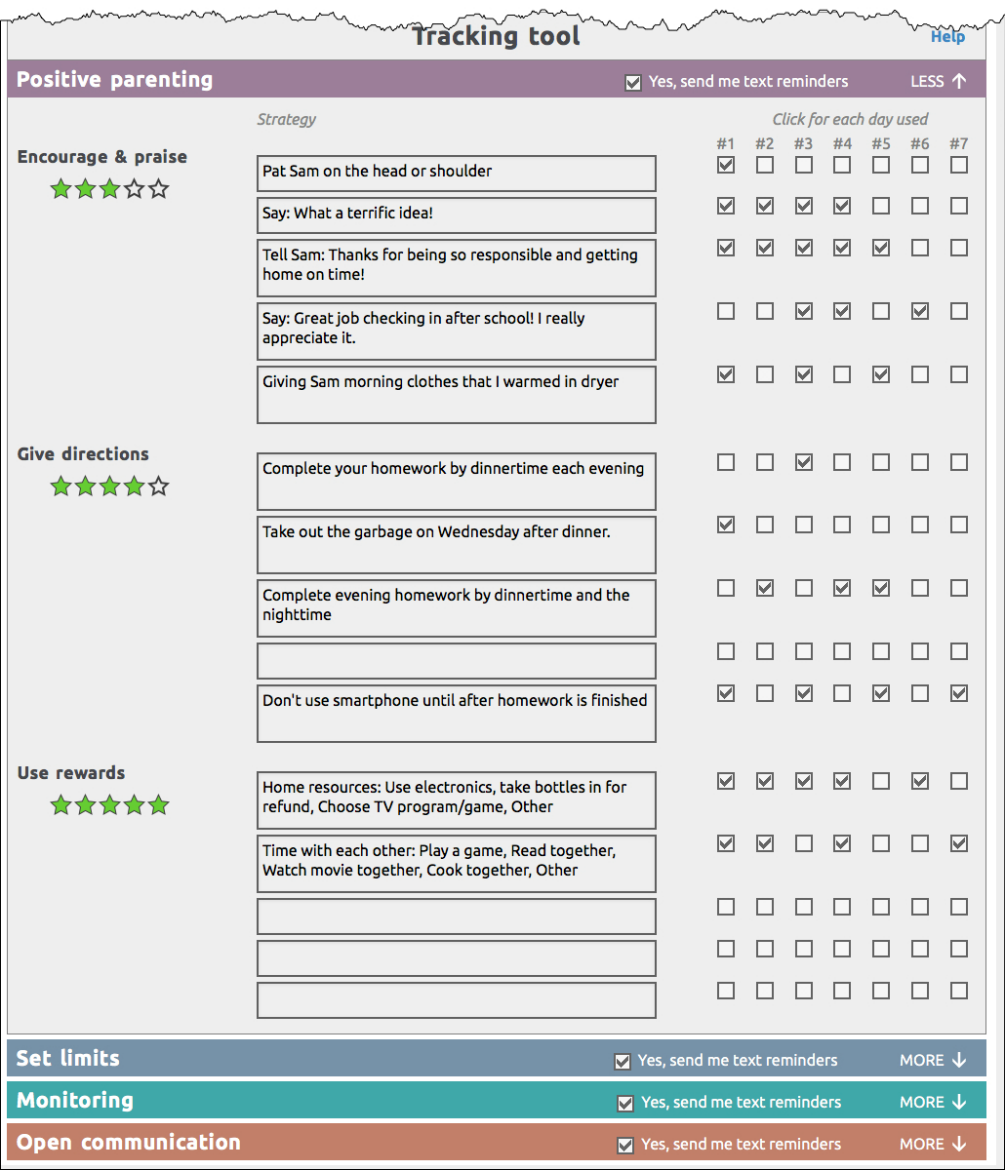
The Family Check-Up Online tracking tool that excerpts strategies (list items) that participants choose to change in each of the 4 skills sessions. It displays an opt-in checkbox for receiving text messages (chosen by type of skill), stars for rating value or helpfulness of each type of skill, and daily practice indicators. Participants can edit and update the contents of this form at any time.

Engagement activities include host videos, dyad videos that model right ways and wrong ways, animations (bear videos) that model right ways, and animated explanations of self-management and problem solving (Figure 5). The program also pushes prompting messages using automated text (SMS) messaging and emails [60]. Figure 6 shows the online tracking tool.

**Figure 7:**
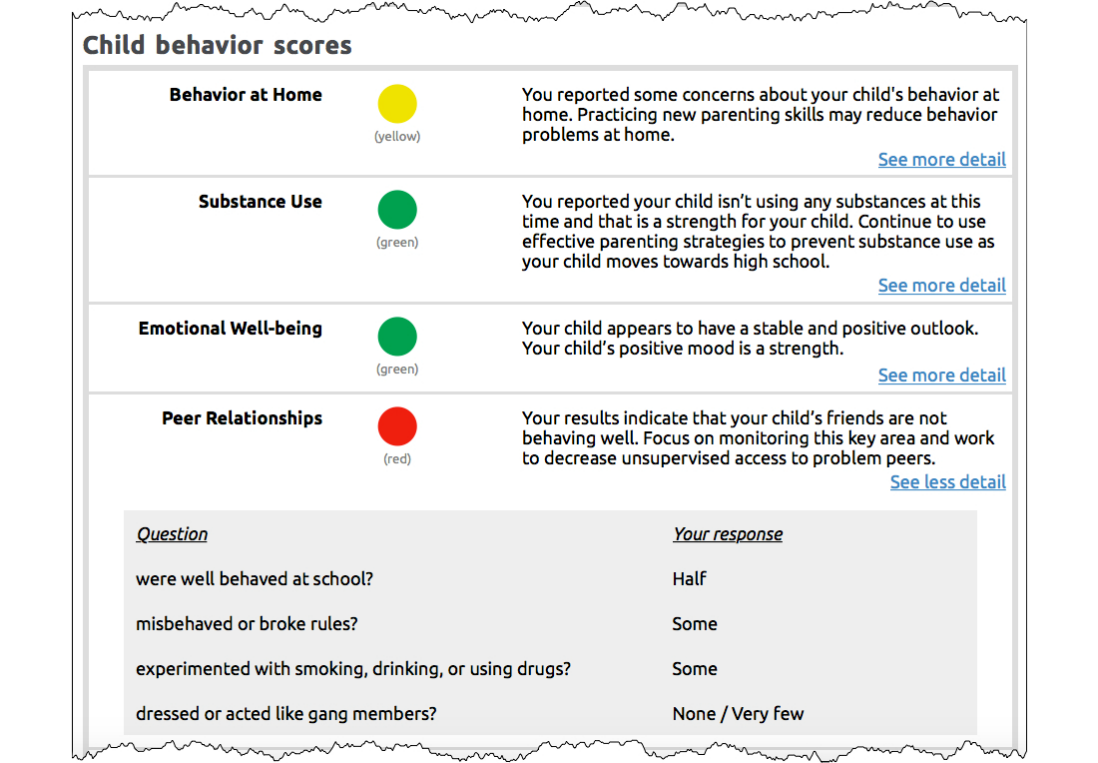
The Family Check-Up (FCU) Online check-up summary report available to participants by clicking on a button located on the FCU home page. A similar report is available to coaches in their administration website. This image shows child behavior scores displayed by color of calculated importance. It also shows drill-down detail (accessed by clicking on the blue text link labeled “See more detail”) listing check-up items and related responses that contributed to the scores.

Other features include a Library (on the Tab menu) that provides articles about relevant topics (eg, cyberbullying, sibling rivalry, and healthy courtship), videos drawn from the skills sessions, and information sheets that can be printed and saved to computer devices for later reference; a Profile (on the Tab menu) that enables participants to update their personal program information, which contains personal information used by the program (eg, names, addresses, passwords, and mobile phone number); and a checkup summary (button on the home page) that helps participants see how they score overall on their checkup assessment and on specific checkup items (Figure 7).

#### Family Check-Up Online Administration Website

**Figure 8:**
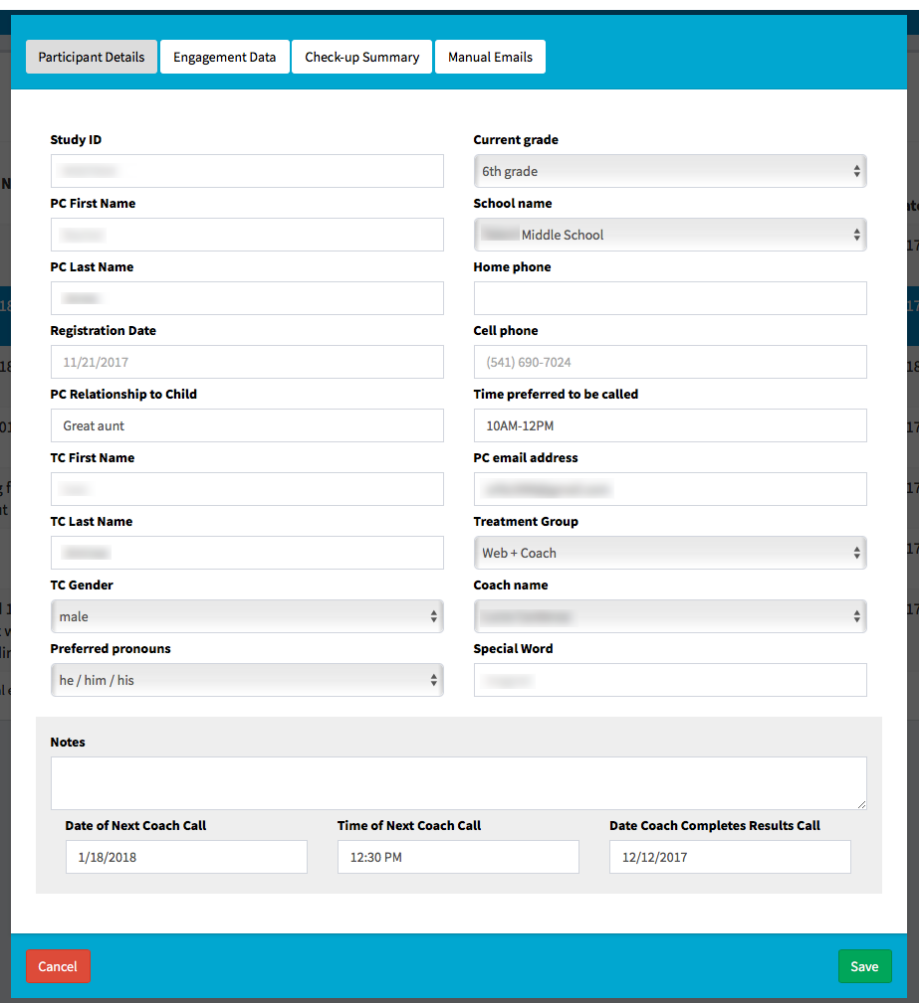
The Family Check-Up Online administration website form used by coaches to describe participant details. It includes fields at bottom of page for jotting down freeform notes and keeping track of key dates and times for coach calls and the check-up results call. ID: identifier; PC: parent caretaker; TC: target child.

**Figure 9:**
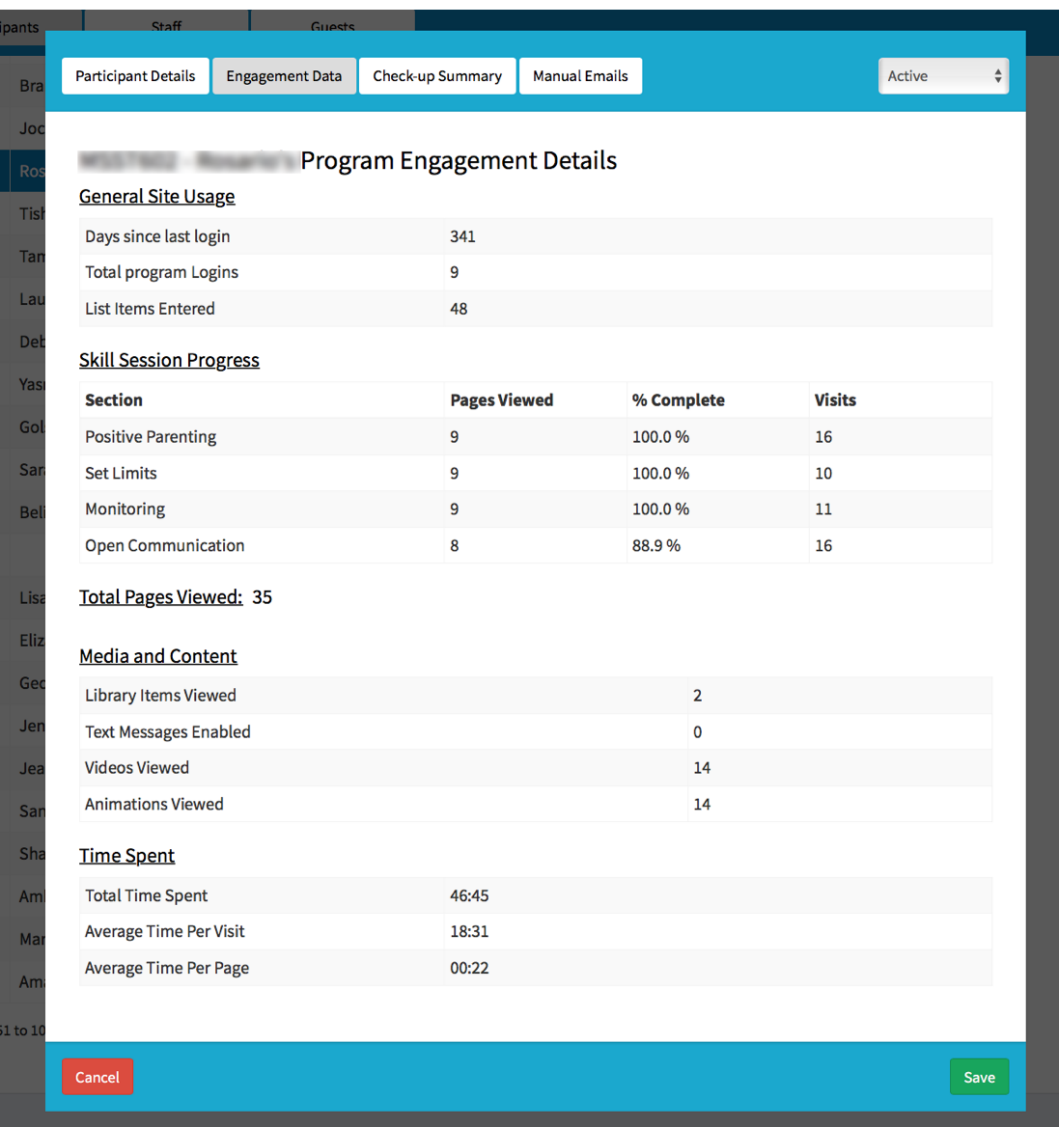
The Family Check-Up Online administration website form for coaches and administrative staff to review measures of participant engagement in using the program, showing data that are collected unobtrusively.

The FCU Online program administration website varies its display of program content on the basis of user credentials. Specifically, study administrators and staff are able to see a list of participants by their name, their unique study identifier, their phone number, their email address, the target child’s name and school, and other descriptive fields. Coaches are able to view only their assigned cases in the coach portal (Figures 8 and 9). Designated guest users are able to review only the features of the website by examining a test case that was created solely for this purpose.

### Randomized Controlled Trial

#### Study Design

**Figure 10:**
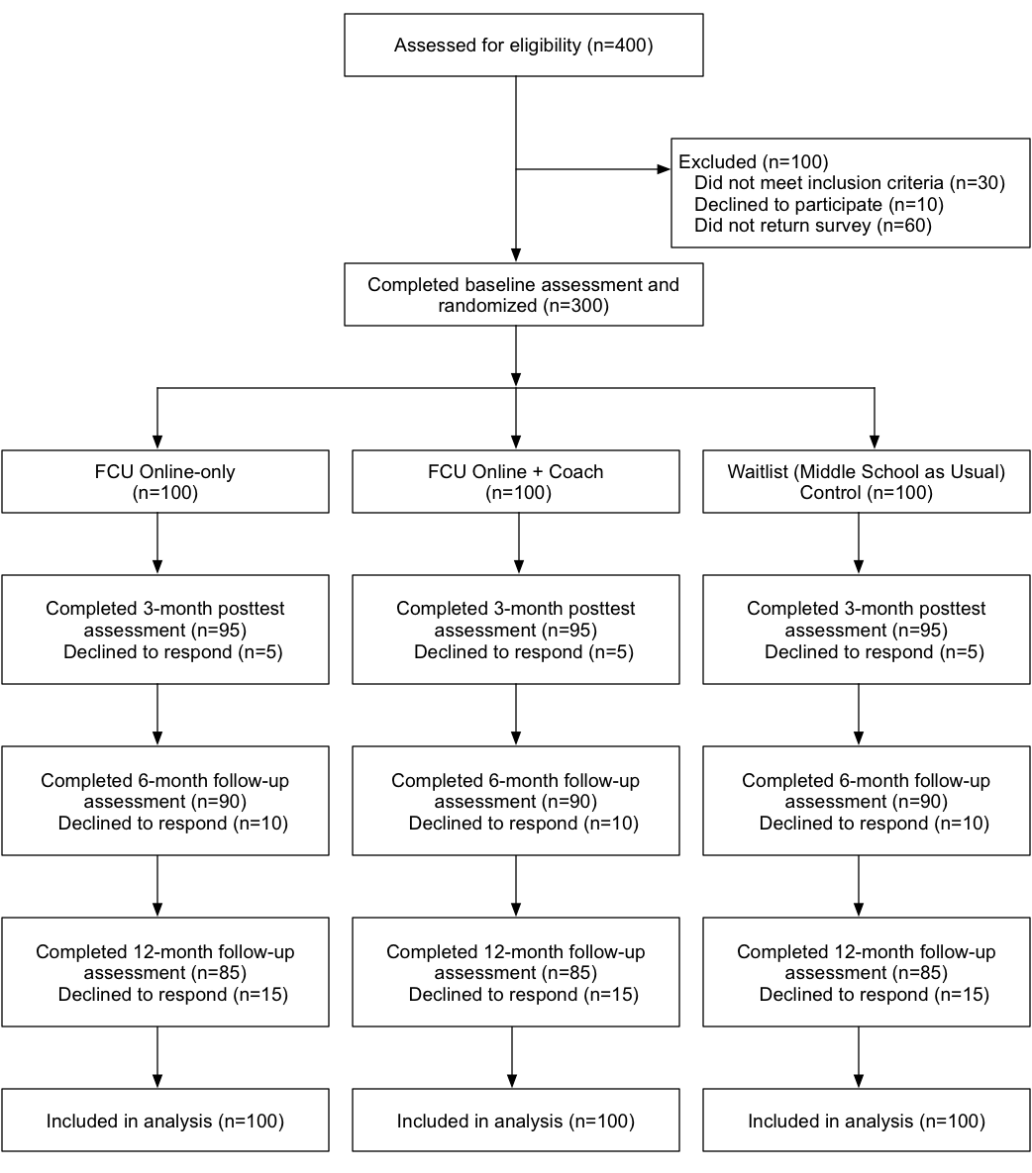
Consolidated Standards of Reporting Trials (CONSORT) diagram of study participants (projected). FCU: Family Check-Up.

In this ongoing study, families meeting eligibility criteria are individually randomly assigned (allocation ratio of 1:1:1) into 3 study conditions: FCU Online-only (n≈100), FCU Online + Coach (n≈100), and a waitlist control condition (middle school as usual; n≈100). Assessments are conducted at baseline, 3 months following randomization (posttest), and at follow-ups scheduled for 6 months following randomization and 12 months following randomization. Figure 10 shows the projected Consolidated Standards of Reporting Trials diagram of study participants.

**Table 1:** Participant engagement activities in the Family Check-Up Online program.

Activity	Function	Examples
List activities	Encourage creation of personal lists to gain insight into their situation	Lists of ways to encourage praise, give directions, and give rewards; household rules; consequences; monitoring skills; school support monitoring skills; active listening skills
Role-playing activities	Practice step-by-step situations and responses	Handling challenging situations; communicating by listening to facts and connecting with feelings
Drag-and-drop activity	Provide an interactive experience to more clearly distinguish between topics	Activity focusing on the difference between helpful and unhelpful ways of giving directions
Online behavior tracking	Web-based tools used to capture participant data over time designed to encourage self-monitoring, to illuminate patterns, and to show progress	Daily tracking of mood ratings and pleasant activities accomplished; these tracked data are also charted online
Wizard/calculator	Tool to help plan schedule	School on-time calculator
Animated tutorials	Animations used to provide an explanation for underlying models for change	Tutorial showing self-management model of trying out new activities, tracking to see if they help, refining them accordingly
Tracking tool	Tool for managing personal practice of recommended strategies and skills	Tool to help monitor activities that are being worked on in each of the major skills areas, ratings for how that practice is going, and ability to edit and update as needed

#### Participant Recruitment and Screening

Recruitment has been completed. The research sample is intended to be approximately 300 families recruited from 8 economically disadvantaged middle schools in Oregon, USA, defined as schools that exceed the state average of 55% of students qualifying for free and reduced-price lunch. We drew 2 schools from urban settings, 4 from suburban settings, and 2 from rural areas in the state. Families in these 3 settings may have different community norms regarding parenting behavior and different degrees of access to mental health services or support for positive parenting. They may also use Web-based resources with different frequency. We are seeking to understand whether community characteristics have an impact on the uptake of an eHealth parenting intervention.

We recruited participants during 2 school years (2016-2017 or 2017-2018) and are currently finalizing our sample. Approximately 150 families of sixth- and seventh-grade students were recruited each year. All families in the designated schools and grades were eligible to participate. Inclusion criteria specified that parents or primary caregivers be legal guardians of the enrolled student and have Web access at home or be willing to access the Web via computers located in the school, public library, or work, and that they have proficiency in English. Families of students with severe developmental disabilities or physical disabilities (eg, autism, genetic disorders, or Down syndrome) were excluded from the study. We expect the ethnicity of the sample to be consistent with that of families in Oregon: about 78% white, 12% Latino, 5% Asian American, and 5% African American.

Institutional review board–approved study procedures took into consideration the privacy protections outlined in the US Family Educational Rights and Privacy Act [62]; University of Oregon Institutional Review Board Protocol Number: 07032014.004).

The recruitment process began with an email sent to parents of sixth- and seventh-grade students from the principal at each school that included a brief description of the study, stated the enthusiastic support of school staff for the project, and included a link to a secure website where interested parents were asked to provide their contact information. A paid research staff member then called all respondents to screen for eligibility, determine parent preference for receiving materials in English or in Spanish, explain the goals of the study, and provide details about participation.

Families who indicated an interest in the study and satisfied eligibility criteria were mailed a packet that included a parent consent form, a youth assent form, the parent and youth surveys, and 4 self-addressed, stamped envelopes so that each consent and survey could be mailed back separately to project staff. This preassessment included standard, widely used questionnaires that ask about the child’s abilities and behavior, parenting practices, family dynamics, family demographics, family health behaviors, and life stressors and took about 30 minutes to complete. One week after this packet was mailed, a research staff member called the parent or caregiver to answer any questions about the consent form or the survey. If the family had not yet returned their surveys, they were reminded to do so as soon as possible.

#### Randomization to Conditions

Once we receive the completed surveys and consent forms, we randomly assign participating families to a condition within the school such that each school will have a similar number of participants in each of the 3 conditions: FCU Online-only, FCU Online + Coach, and a waitlist control. Spanish-speaking parents who previously indicated comfort reading in English are randomly assigned to 1 of these conditions; parents who indicated greater comfort reading in Spanish are assigned to a nonexperimental telehealth treatment condition that receives print materials in Spanish and interacts with a coach in Spanish.

In the FCU Online-only condition, participants receive a welcome email with an explanation about the website and instructions for logging in. Once they log in to the FCU Online website with their credentials, participants are able to complete the FCU assessment, receive feedback, and then access the skills session website, where they are provided with online tools to support their parenting in areas identified as challenges. These tools include videos, animated videos, parenting tips, and interactive activities (see Program Components section of this paper). Parents are also given the opportunity to practice parenting skills and track their progress. Parents can receive text messages that prompt them to try out new skills learned from the website. Parents can log in as often as they like and interact with any of the parenting skills sessions on the website.

In the FCU Online + Coach condition, participants receive a welcome email with an explanation about the website, instructions for logging in, and the name and email address of the coach who will be working with them. Participants in this condition log on to the same FCU Online program and follow the same procedure made available to participants in the online-only condition. However, they are also assigned a family coach who calls them at least two times to help establish goals, talks them through their results, offers support, and helps motivate parents to improve parenting practices. These coaching calls are intended to be brief and focused, and to last as long as necessary, but typically for less than 30 minutes. Coach calls are scheduled based on a family’s availability, and they may be initiated by either the coach or parent.

In the waitlist control (middle school as usual) condition, participants receive an email thanking them for their participation and letting them know that project staff will next contact them in 3 months, when they complete another questionnaire.

### Measures

Families in all 3 conditions are mailed a follow-up questionnaire at 3 months, 6 months, and 12 months that is identical to the preassessment in order to assess changes in constructs over time (see Table 2). Each follow-up questionnaire is estimated to require 30 minutes to complete. The 3-month follow-up questionnaire for parents in the 2 intervention conditions also includes a 2-page website feedback survey that requires approximately 10 minutes to complete. All families receive remuneration for their time: US $100 for completing the baseline assessment and US $100 for completing each of the 3 follow-up assessments, for a total of $400. Families can also earn a US $50 bonus if they complete all 4 assessments.

#### Sociocultural Contexts and Resources

Family resources and contextual stressors are assessed. Background variables are obtained from primary caregivers by using our project-generated Demographic Questionnaire, which includes queries about family income, parents’ occupational status, education, marital status, living situation, and parenting arrangements, if any. Parent and child physical health are also assessed, as is social service use. In addition, parental emotional well-being (depression and anxiety), substance use, and relationship satisfaction are assessed.

Parent physical health is assessed with 3 items regarding height, weight, and perception of general health. Child physical health is assessed with 5 items taken from the Child and Family Center Student Survey (CFCSS) [63] regarding height, weight, perception of body size, consumption of soda and sweet drinks, and daily exercise. In addition, the family’s engagement in healthy food choices and physical activity is assessed with a 31-item modified version of the Family Health Behavior Scale [64].

**Table 2:** Assessment timeline.

Constructs			Baseline	Posttest (3-month assessment)	Follow-up (6- and 12-month assessments)
**Family sociocultural contexts and resources**					
	Family income		P^a^	P	P
	Parent’s occupational status		P	P	P
	Parent education		P	P	P
	Parent marital status		P	P	P
	Living arrangements		P	P	P
	Parenting arrangements		P	P	P
	Parent physical health		P	P	P
	Child physical health		P	P	P
	Family health behaviors		P	P	P
	Parent anxiety and depression		P	P	P
	Parent substance use		P	P	P
	Parent relationship satisfaction		P	P	P
	Service use		P	P	P
**Parenting skills and behavior**					
	Setting limits		P	P	P
	Monitoring of peer relationships		P, C^b^	P, C	P, C
	Monitoring of family routines		P	P	P
	Positive parenting		P, C	P, C	P, C
	Parental involvement in child’s school		P	P	P
	Parent self-efficacy		P	P	P
**Youth adaptation and family outcomes**					
	**Youth problem behavior**				
		Child’s effortful control	P, C	P, C	P, C
		Youth adjustment to school	P, C	P, C	P, C
		Strengths and Difficulties Questionnaire	P, C	P, C	P, C
		Child substance use	P, C	P, C	P, C
		Child attitude about substance use	C	C	C
		Child association with deviant peers	P, C	P, C	P, C
	**Family relationships**				
		Family conflict	P, C	P, C	P, C
		Positive family relationships	P, C	P, C	P, C
		Positive family support	P, C	P, C	P, C
**Family engagement**					
	Program use^c^		P	P	P
	Website satisfaction		—	P	—
	Coach calls^d^		P	P	—

^a^P: parent.

^b^C: child.

^c^Program use was monitored automatically and unobtrusively by the intervention website over the course of the project period.

^d^A coach phoned participants in the FCU Online + Coach condition at least two times in the period between baseline and posttest.

Parental emotional well-being is assessed using the 2-item Patient Health Questionnaire depression screener [65] and the 2-item Generalized Anxiety Disorder Scale screener [66]. Parents’ use of tobacco, alcohol, and marijuana is evaluated with a brief 3-item version of the Parent Substance Use Questionnaire [12]. Parental relationship satisfaction is assessed using the 4-item screening version of the Dyadic Adjustment Scale short form [67,68]. Finally, service use is assessed using 7 items adapted from the Services Assessment for Children and Adolescents [69] regarding mental health, medical, or school services received in the past year by the child, primary caregiver, or other household member.

#### Parenting Skills and Parenting Behavior

Parental skills and behavior are assessed using both parent- and child-report measures. Parents report about setting limits with 7 items excerpted from the Parenting Children and Adolescents measure, an unpublished older-child version of the Parenting Young Children measure [70]. Monitoring of peer relationships, monitoring of family routines, and positive parenting is measured with 21 items adapted from the Parent Interview [71,72]. Youth also report about positive parenting and parental monitoring of peer relationships with 13 parallel items adapted from the CFCSS [63]. Parental involvement in the child’s school and parent self-efficacy are also assessed through parent self-report via 6 items from the Parent Involvement Scale [73] and 8 items adapted from the Parenting Task Checklist [74].

#### Youth Problem Behavior and Adaptation

Parents and youth report about the teen’s effortful attention control via an 8-item subscale of the Early Adolescent Temperament Questionnaire [75,76]. Parents and youth also report about the youth’s adjustment to school by using 5 items adapted from a measure of school participation [57]. Problem behavior is assessed through several modalities. Parents and youth report about problem behavior using the 26-item Strengths and Difficulties Questionnaire [58]. Child substance use is assessed with 3 parent-report items about the frequency of their child’s tobacco, alcohol, and marijuana use and with 4 child-report items about tobacco, alcohol, and marijuana use in the past month and about riding in a car with someone under the influence. Children also report about the perceived difficulty of obtaining tobacco, alcohol, or marijuana (3 items) and their attitudes regarding these substances (3 items), adapted from the CFCSS [63]. Child association with deviant peers is also assessed via parent and child report with 4 items adapted from the Peer Affiliation and Social Acceptance measure [77].

#### Family Relationships

Parents and children both report about family conflict, positive family relationships (4 items), and positive family support (3 items) [78].

#### Family Engagement

Family engagement with the intervention is assessed in three ways. First, we look at participant use of the eHealth intervention. Each participant in each of the 2 intervention conditions determines how often and for how long they interact with the program, which is assessed unobtrusively by the program. Following an approach we used in earlier research on eHealth interventions [28,79,80], we created a composite measure of program engagement defined as the product of the *z* score transformations of the mean of (1) the overall duration of program visits, and (2) the overall sum of the number of visits. These measures also allow us to assess the extent to which each participant used each module in the program by using a more detailed assessment of engagement activities (eg, reviewed videos and animations, opened online documents, created personal lists, or tracked practice activities).

Second, consumer satisfaction with the website is assessed at the time of the 3-month posttest using a measure developed for parent-training programs [81], which includes satisfaction with content and delivery of the model and factors related to uptake and use of the information. We adapted it for this study to also assess barriers parents may face in completing an eHealth intervention (eg, time or computer equipment).

Third, in the FCU Online + Coach condition, family engagement is assessed via the number of contacts with a coach, total minutes of contact with a coach, and overall therapy dosage.

#### Implementation Assessment

During project year 5, we will assess our effort to encourage the continued implementation of the FCU Online program in schools that participated in the research project. We will train school personnel in both versions (FCU Online-only and FCU Online + Coach) and problem solve with the school to ensure successful uptake, and work with the school to identify families for the intervention by using natural school indicators of success (eg, attendance, behavior referrals, and grades). We will then assess uptake of the intervention by interviewing teachers and administrators about their usage. In addition, we’ll use our measure of successful uptake of family-centered, school-based interventions, the Family-School-Wide Evaluation Tool [82], based on the widely used School-Wide Evaluation Tool assessment for evaluating uptake of positive behavior support programs in schools [83,84]. This will occur at the end of year 5 after the schools have had a chance to implement the program throughout the year.

### Formative Research Process

At multiple points in the iterative development process, we have gathered information from potential users about what was working in the program and what needed to be adapted or reformulated. The first of these focus groups included 6 parents and the dean of students from a participating middle school. Three other focus groups included various school staff from 4 schools (2 suburban and 2 rural). The first included 2 principals, 2 vice principals, and 1 school counselor; the second included 1 principal, 2 deans of students (1 of whom was also called a family liaison staff), and 1 counselor; and the third included 1 principal, 1 counselor, and 1 behavioral specialist. We integrated the feedback from these groups into the development and design of the program (eg, enable users to go back and retake the FCU assessment, provide more tips and the shorter the better, have children looking at mobile phones in the pictures so the images appear more accurate and up-to-date, allow different credential levels on the administration site).

Next, we conducted usability testing with 5 participants to examine the acceptability and feasibility of the program. Usability testers met individually with a research staff member in 90-minute sessions during which they interacted with portions of the FCU Online program while testers used think-aloud techniques to describe their ideas and thoughts. Usability test participants were also asked to complete the 10-item System Usability Scale [85] to examine the acceptability and feasibility of the program. Items in this scale include “I think that I would like to use this website frequently” and “I thought the website was easy to use.” We then used these qualitative and quantitative data to further improve the design of the program.

Finally, we conducted a pilot study with 7 participants in either the FCU Online-only or the FCU Online + Coach condition. Parents were then given 2 weeks to use the website and meet with a coach (if applicable). Next, parents provided verbal feedback about their experience with the assessment process in general, and with the website specifically, during a debriefing interview with project staff members. This feedback was used to further improve the surveys and program logic.

### Data Analysis

Families are randomly assigned to a condition and will be the unit of analysis for all models. Mixed-effects analyses will be based on a hierarchical linear modeling approach in which students are nested within schools; primary outcomes are nested within individual students at level 1 of the model; and between-participant predictors (fixed effects), such as treatment condition and child and parent demographics, will be examined at level 2. This approach will (1) account for the correlated within-participant errors created by nesting of repeated measurements within study participants, (2) allow us to examine longitudinal trajectories within a unified and flexible framework that also facilitates examination of potential moderating and mediating variables, and (3) enable us to test for potential dependencies (school-level effects) in the data. For each of the 3 pairwise contrasts between conditions, we will examine intervention effects by modeling longitudinal trajectories across time with mixed-effects models using SAS PROC MIXED (SAS Institute) or Mplus software (Muthén & Muthén).

Using an intent-to-treat approach with 100 participants per condition, with alpha set to .017 (to adjust for multiple contrasts), there is sufficient power (>.80) to detect a condition effect of Cohen *d*=.42 or larger (moderately small effect size) between either intervention condition and the control group on primary outcomes, which include effective parenting skills and reductions in child problem behavior. Previous FCU efficacy trials have demonstrated medium to large effects for tobacco use, alcohol use, cannabis use, antisocial behavior, and arrest rates [15,21].

## Results

This project was funded in 2015 and the research project period is scheduled to be completed in 2020. Participant recruitment was completed in spring 2018 and initial assessment is ongoing. Follow-up assessments will not be completed until 2019.

## Discussion

### Overview

This paper describes the innovative FCU Online eHealth intervention randomized controlled trial for parents of middle school children. Our report focuses on the background, research design, and intervention components of a trial that will develop and test the usability, feasibility, and efficacy of an eHealth version of the FCU program that targets middleschool children. The rigorous study design will allow for comparisons of two versions of the FCU Online program (FCU Online + Coach, FCU Online-only) and the waitlist control condition.

### Strengths and Limitations

A fundamental strength of the FCU is that it is scalable at multiple levels, depending on the barriers and resources available (family resources and school resources). For schools, barriers in staffing and coach support may prohibit use of the FCU Online + Coach, and these schools can use the FCU Online-only version, which requires limited staffing support to provide access to families. Barriers for families include time, transportation, and internet access. The FCU Online program can be delivered to families in their home and on their own schedule. The website is accessible by phone and can also be used “on the go.” Schools may also provide computer access to enable families to complete the program. This provides a high-reach, scalable approach to help families that is accessible to urban, suburban, and rural communities.

Another strength of this study is the use of multiple urban, suburban, and rural schools throughout the state of Oregon. This diverse population will allow us to examine rural versus urban participation and consumer satisfaction. We plan to implement the FCU Online program in 1 model school at the end of the study, which will provide additional information about dissemination.

Our development approach is also a strength of this study. Specifically, our use of an integrated technology architecture and a shared database facilitates data sharing and consistent programming procedures for the FCU Online website, its administration website, and its coach portal. Similarly, our use of an iterative formative research development process helped confirm program functionality and refine the program’s user experience design and user interface.

A potential limitation is the financial remuneration of the maximum amount of US $450 that participants are scheduled to receive in this efficacy trial for time and effort spent completing the assessments. We believe that this level of remuneration is equivalent across conditions and therefore it should not differentially affect groupwise outcomes. Moreover, it is contingent upon assessment completion rather than participation in the intervention. However, given the likelihood that the FCU Online program would not be implemented in the real world with such significant financial consequences, it will be important to assess the FCU Online program within a more practical context. In addition, previous research on the FCU has demonstrated prevention effects emerging over long-term, multiyear follow-up. As such, potential long-term prevention effects of the FCU may not be detected within the 1-year follow-up period of this study. Another possible limitation is that participants using the current version of FCU Online program must be proficient in English.

### Future Directions

Programs such as the FCU Online represent an important next-generation direction in delivering behavioral health programs to parents and caretakers of school-aged children. Previous researchers and clinicians have been working to integrate evidence-based behavioral health prevention and intervention programs (also termed mental health programs) into schools for more than three decades (eg, [86,87]). Nearly all these attempts identified a consistent set of barriers when moving from “hothouse” efficacy demonstrations to the real-life frontiers of community educational settings and service providers. These barriers primarily include lack of resources (particularly in rural settings); stigma and parental resistance associated with behavioral health screening and diagnostic methods [88]; competing responsibilities of intervention staff; lack of support from school administrators and teachers, who often have no exposure to or training in behavioral health practices that are evidence based; difficulty in engaging families; and administrative and staff turnover, which creates a tremendous and ongoing staff training problem. The fact that many evidence-based practices are not flexible in terms of allowing shorter sessions and briefer interventions, and that most are developed for single issues (eg, anxiety, depression, or oppositional defiant disorder), makes it difficult to integrate various behavioral health programs [57,87,89], which creates additional barriers to uptake in real-life community settings.

In the context of all these barriers, schools are faced with increasing challenges, such as climbing rates of mental health issues, high rates of behavioral problems, children exposed to trauma, and school violence [90,91]. The FCU Online program has the potential to help address many of the aforementioned barriers that more traditional school-based behavioral health implementation strategies have yet to solve. For example, strengths of the FCU Online include its ability to be used within any behavioral health service delivery model or strategy (eg, school-only, school plus community behavioral health clinicians, and school-based health clinics), the ability of parents and clinicians to titrate (ie, use indicated modules or the entire program), and the ability to select families for intervention in a nonstigmatizing manner (ie, nonuse of *Diagnostic and Statistical Manual of Mental Disorders* diagnostic labels). The FCU includes a contextualized assessment of known family, child, and parenting constructs associated with behavioral health and educational outcomes (common elements approach); engagement of parents with multiple and parent-preferred levels as opposed to the more usual face-to-face–only strategies; presentation through multiple methods (eg, human video; video animations; text, graphs, and tools; exercises and forms; and additional literature) of evidence-based parenting methods and exemplars that are not confounded by clinician talent and training; and low response cost for schools to integrate at whatever level their desire or resources allow.

In addition, it remains for additional research to demonstrate the extent to which adding complementary program content (eg, stress management skills training, healthy eating) aimed at parents might enhance impact and whether benefits might accrue from adding online content for children. For example, embedding content from an evidenced-based curriculum, such as Coping Power, that includes both child and parent components into FCU Online might enhance outcomes over time [92]. Additional research on implementation and sustainability of effective interventions might explore whether targeted eHealth interventions for families, such as the FCU Online program, could be but one element of a multicomponent, school-based mental health program that provides access to engaging internet-based resources and tools [93,94]. Future analyses (following the examples set by the recent review by Finan et al [95] and the study by Heinrichs [96]) should also examine the possible impacts of different amounts of behavioral health prevention dollars used (1) to incentivize recruitment and assessment completion and (2) to encourage the practice of program strategies that might sustain treatment effects long after research payments are no longer an option.

Plans for future development of the FCU Online program include more-varied approaches and reporting to accommodate multiple parents and caretakers per child, multiple children within a participating family, expansion of program content for use by Spanish-speaking users, and expansion to health-related topics (eg, obesity management and prevention, and health promotion).

## Appendix

Multimedia Appendix 1 Peer-reviewer report: summary statement generated by peer reviewers in NIDA Study Section.

## Abbreviations

CFCSS: Child and Family Center Student Survey
eHealth: electronic health
FCU: Family Check-Up
SMS: short message service
