# Supplementary material for: The Family Check-Up Online Program for Parents of Middle School Students: Protocol for a Randomized Controlled Trial
Source: JMIR Res Protoc. 2018 Jul 18;7(7):e11106. doi: 10.2196/11106 (PMC6070726; doi:10.2196/11106)
Supplement: Multimedia Appendix 1 [file resprot_v7i7e11106_app1.PDF]

SUMMARY STATEMENT
(Privileged Communication)

PROGRAM CONTACT:
Belinda Sims
301-402-1533
bsims@mail.nih.gov

Release Date: 06/30/2014

Application Number: 1 R01 DA037628-01A1

Principal Investigators (Listed Alphabetically):
SEELEY, JOHN R PHD
STORMSHAK, ELIZABETH A PHD (Contact)

Applicant Organization: UNIVERSITY OF OREGON

Review Group: PDRP
Psychosocial Development, Risk and Prevention Study Section

Meeting Date: 06/09/2014
Council: OCT 2014
Requested Start: 10/01/2014

RFA/PA: PA13-302
PCC: CV/BSV

Project Title: Prevention of Substance Use in At-risk Students: A Family-centered Web Program

SRG Action: Impact Score: 10 Percentile: 1
Next Steps: Visit http://grants.nih.gov/grants/next_steps.htm
Human Subjects: 30-Human subjects involved - Certified, no SRG concerns
Animal Subjects: 10-No live vertebrate animals involved for competing appl.
Gender: 1A-Both genders, scientifically acceptable
Minority: 1A-Minorities and non-minorities, scientifically acceptable
Children: 1A-Both Children and Adults, scientifically acceptable
Clinical Research - not NIH-defined Phase III Trial

Project Year	Direct Costs Requested	Estimated Total Cost
1	496,621	738,035
2	477,685	709,894
3	499,892	742,896
4	499,278	741,984
5	497,552	739,419
TOTAL	2,471,028	3,672,227

ADMINISTRATIVE BUDGET NOTE: The budget shown is the requested budget and has not been adjusted to reflect any recommendations made by reviewers. If an award is planned, the costs will be calculated by Institute grants management staff based on the recommendations outlined below in the COMMITTEE BUDGET RECOMMENDATIONS section.

1R01DA037628-01A1 STORMSHAK, ELIZABETH

RESUME AND SUMMARY OF DISCUSSION: This application proposes to develop and test the efficacy of an online version of the Family Check-Up (FCU) for schools that parents can use either at the school or at home. The expected overall impact is high and the proposed research has the potential to advance knowledge about the efficacy of a web-based version of the Family Check-UP for families of middle school youths that can reduce later risk of problem behaviors and substance use. The reviewers noted that this submission has been responsive to the previous review and the application has improved. The use of the web to deliver family centered intervention in schools, the use of online versus online plus coach conditions, and examining the targeted intervention mechanisms as mediators of the intervention main effect are considered innovative. During the discussion, the reviewers indicated that there are many strengths of the proposed research including the study design, intervention components, the outcome and mediator assessments of youth problem behavior, parenting skills, involvement and family relationships, the randomization procedures, and the three month assessment of substance use. Overall, the reviewers agree that the proposed research is highly significant and innovative and will likely have a high impact in the area of understanding effective interventions for adolescents that reduce risk behavior and substance use during the transition to high school.

DESCRIPTION (provided by applicant): School-based, family-centered interventions for reducing substance use in adolescence have been shown to be highly effective and result in reductions in problem behavior, achievement problems, depression, and substance use over time. Although these interventions have been shown to be effective in efficacy trials across the United States, few schools have the resources to implement these interventions without substantial support. Barriers such as low staffing, poorly trained staff, and limited resources prevent the successful uptake of family-centered interventions in schools, yet most school systems are supportive of enhancing family-school partnerships. The proposed study will build upon our successful program of research by developing and testing the efficacy of an online version of the Family Check-Up (FCU) for schools that parents can use either at the school or at home. The FCU-Online will incorporate the successful components of the original FCU, such as a strengths-based assessment that is norm referenced, feedback using motivational interviewing, and follow-up modules that focus on enhancing parenting skills and family management in early adolescence. In the proposed research, 300 families will be randomly assigned to receive middle school as usual, the FCU-Online web/mobile only, or the FCU-Online web/mobile+coach version. Research on the delivery of mental health interventions via the Internet suggests that a coach or "in person" contact enhances outcomes; however, programs that require no coaching or in-person contact may have a larger public health impact. A coach version of the intervention will be compared with an online-only version that requires no staffing at schools. We will test the efficacy of this online version with respect to changing key parenting constructs, such as positive parenting and parental monitoring. We predict that changes in parenting will lead to reductions in risk behavior, such as problem behavior at school and substance use. This research will significantly contribute to our understanding of effective interventions for adolescents that reduce risk behavior and substance use during the transition to high school, and it can significantly contribute to the overall uptake and dissemination of family- centered interventions in schools.

PUBLIC HEALTH RELEVANCE: We will examine the hypothesis that a brief, tailored, web-based family-centered intervention provided to families of middle school youths can reduce behavioral risk, enhance parenting skills, improve family climate, and improve child outcomes. If the intervention is successful, it could (a) be cost-effectively implemented in middle schools across the United States, (b) significantly improve the adjustment of children and families during the middle school years, and (c) reduce later risk of problem behavior and substance use for adolescents. The study will contribute considerably to our understanding of how to best implement evidence- based practices in schools and use web-based interventions to reach at-risk youths and their parents, with the potential to reach large populations of families and thus have a positive impact on public health.

CRITIQUE 1:

Significance: 1
Investigator(s): 1
Innovation: 1
Approach: 1
Environment: 1

Overall Impact: The proposed study seeks to develop a web-based implementation of the Family Check-Up intervention for schools that parents can use either at school or at home. The FCU-Online intervention would incorporate the successful components of the original FCU, including a strengths-based assessment that is norm referenced, feedback using motivational interviewing, and follow-up modules that focus on enhancing parenting skills and family management in early adolescence. Under ARRA funding, the researchers developed a web-based dissemination/ implementation tool for practitioners including video examples of parenting and worksheets. The proposed study would adapt and test this web-based intervention for use directly by families. In the proposed intervention test, 300 families of kids in the bottom third on attendance and grades would be randomly assigned to one of three arms: middle school as usual, the FCU-Online web/mobile only, or the FCU-Online web/mobile+coach. Year 1 employs focus groups with school staff and parents to inform intervention design. Years 1-2 for design and testing, implementation in year 3 with assessments and follow-ups in years 3-5. Students will be recruited across six middle schools, and will be randomly assigned to the two intervention and school-as-usual control group, as stratified by gender. Although recruited by school, the interventions are at the family level. The study is of high scientific and public health significance, addressing two major problems currently vexing prevention science: how to go to scale, and how to improve parent engagement in interventions? Thus, the development of effective school-family interventions that are avidly taken up by families is a highly significant public health area. It is precisely this sort of study---that seeks to facilitate the implementation of existing tested, effective, but somewhat challenging to implement interventions---that is needed at this time in prevention. Overall, this is an exceptionally well-planned study that has been thought through in great detail. The intervention design, the pre-test/intervention building phase, the assessment battery, implementation plan, and analysis plan are all thorough and reasonable. The team has extensive experience in intervention design and web-based adaptation of interventions. The revision was highly responsive and this reviewer has no concerns regarding this highly significant and well-designed study.

1. Significance:

Strengths

- The development of effective school-family interventions that are avidly taken up by families is a highly significant public health area.
- It is precisely this sort of study---that seeks to facilitate the implementation of existing tested, effective, but somewhat challenging to implement interventions---that is needed at this time in prevention.
- Contributions of this study over-and-above existing research are well described.

Weaknesses

- None noted.

2. Investigator(s):

Strengths

- Dr Stormshak has extensive experience in substance use preventive intervention design and implementation in general and the Family Check-Up in particular. Drs. Seeley and Danaher have extensive experience in the design, development and evaluation of web-based behavioral interventions. The remaining members of the project team each add significant, meaningful contributions to the proposed study.
- Personnel seem to be budgeted at sufficient FTE to effectively implement the proposed study.
- Consultants (Drs. Dishion and Lochman) provide additional depth of experience in the FCU and in school-based preventive intervention.

Weaknesses

- None noted.

3. Innovation:

Strengths

- The innovations are: the use of the internet to deliver family-centered intervention in schools; the use of online only vs. online + coach conditions, tests the feasibility of a family-centered, web-based program to reduce problem behavior in adolescence and examining the targeted intervention mechanisms as mediators of intervention main effects. Examining mediational mechanisms of an intervention is good science, however, it happens rarely enough to qualify as an innovation.

Weaknesses

- None noted.

4. Approach:

Strengths

- Overall, this is an exceptionally well-planned study.
- The study design and assignment strategy make sense: Year 1 employs focus groups with school staff and parents to inform intervention design. Years 1-2 for design and testing, implementation in year 3 with assessments and follow-ups in years 3-5. Students will be recruited across six middle schools, and will be randomly assigned to the two intervention and school-as-usual control group, as stratified by gender.
- Under earlier ARRA support a web site was developed for dissemination and implementation of the FCU intervention as implemented by practitioners. Thus, the infrastructure is in place for this to be adapted for parent implementers and then tested for the efficacy of this web-based approach as is proposed in the proposed study.
- The intervention components, and their web/mobile delivery are well thought out, and build logically from the existing FCU intervention. The “Coach” condition is meaningfully designed and provides an interesting test against the web-based only condition.
- The outcome and mediator assessment package consists of tested, reliable and valid measures of youth problem behavior, parenting skills, involvement and family relationships. In addition, school-record data, including grades, attendance, and discipline referrals, will be gathered from schools.
- Letters of support are provided from the superintendents of school districts from which the participating student sample will be drawn.

- The analysis section anticipates missing data appropriately, and anticipates the need to examine differential attrition by intervention condition.
- The application notes that even though this is a family based intervention (and they do not expect intraclass (within school) clustering) they will none-the-less test for this possibility in analyses.

Weaknesses

- None noted.

5. Environment:

Strengths

- The University of Oregon & Prevention Science Institute (within the College of Education) in addition to the co-investigators' site of the Oregon Research Institute provide excellent intellectual and research support for the implementation of the proposed study.

Weaknesses

- None noted.

Protections for Human Subjects:

Acceptable Risks and/or Adequate Protections

- Reasonable consideration of risks and reasonable procedures for minimizing potential risks.

Data and Safety Monitoring Plan (Applicable for Clinical Trials Only):

Acceptable

- Data security and adverse event reporting protocols in place.

Inclusion of Women, Minorities and Children:

G1A - Both Genders, Acceptable

M1A - Minority and Non-minority, Acceptable

C1A - Children and Adults, Acceptable

- Parents and their middle-school children, ethnicity of the sample is expected to be consistent with that of families in Oregon, with about 78 percent White, 12 percent Latino, 5 percent Asian American, and 5 percent African American. Projected equal distribution of gender.

Vertebrate Animals:

Not Applicable (No Vertebrate Animals)

Biohazards:

Not Applicable (No Biohazards)

Resubmission:

- Highly responsive to the initial round of reviews. I have no further concerns.

Resource Sharing Plans:

Acceptable

Budget and Period of Support:

Recommend as Requested

CRITIQUE 2:

Significance: 1
Investigator(s): 1
Innovation: 1
Approach: 1
Environment: 1

Overall Impact: This application has the potential for high public health significance. The goal to reduce substance use and risk behavior in adolescence is important. The design is elegant, the intervention has already been shown to be effective and thus is well suited to be adapted for a web/mobile version. Careful attention has been devoted to the development phase with an iterative process with focus groups followed by a small pilot study. Recruiting students in the bottom third in grades and attendance will likely yield a sample that needs such an intervention the most. The application is highly significant and innovative, the methods are sound with clear rationale for the different phases, the investigative team is top notch, and the environment is excellent. Overall, this is of high impact to the field and to high risk families who may benefit from such an intervention the most.

1. Significance:

Strengths

- Focus on reducing substance use and risk behavior in adolescence is of high public health significance.
- Goal to develop and evaluate the effectiveness of a web-based intervention using the Family Check-Up, a theory driven intervention that has been shown to increase positive parenting and reduce problem parenting practices, has been effective in reducing substance use and behavior problems, promoting transition from middle to high school, and has been tested in diverse samples. Creating a web version of this intervention is likely to increase the public health impact by making the intervention accessible to large numbers of parents of adolescents.

Weaknesses

- None noted.

2. Investigator(s):

Strengths

- This is an excellent investigative team ideally suited to this application. Dr. Stromshak has been the principal investigator of previous iterations of the FCU, Drs. Seeley and Danaher has experience with web-based interventions, Drs. Dishion and Lochman will serve as consultants.

Weaknesses

- None noted.

3. Innovation:

Strengths

- Developing and testing a web version of the FCU is innovative and important. Targeting high risk youth using school based methods is also innovative. The inclusion of a coach vs. non-coach condition is innovative and will likely yield useful information regarding effectiveness. The iterative development process is well reasoned and is innovative.

Weaknesses

- None noted.

4. Approach:

Strengths

- Students will be recruited from six middle schools in Oregon. Clustering of participants within schools will now be examined in data analysis and potential school level effects will be modeled. This is a significant change from the previous application and is now viewed as a strength.
- The timeline is now appropriate and there are no concerns that investigators will be able to complete project tasks in the timely fashion.
- The procedure for randomization has been clearly articulated.
- Teacher and youth reports will be collected as is appropriate.
- Treatment usability by mothers and fathers will be examined provided adequate number of fathers.
- Parent success in learning the new skills will be assessed with repeated assessments at 3 months, 6 months, and 1 year.
- A 3-month window will be used for assessment of substance use and this is appropriate.
- Middle school students (n = 300) will be recruited and assigned to one of 3 conditions: web/mobile intervention with coach, without coach, and a control group. The recruitment and retention strategies are well described and appropriate. The focus on students who are in the bottom third with regard to grade and attendance is also appropriate given the goals of this application.

Weaknesses

- None noted.

5. Environment:

Strengths

- The Prevention Science Institute is well suited to this project. All necessary resources are available.

Weaknesses

- None noted.

Protections for Human Subjects:

Acceptable Risks and/or Adequate Protections

- Random assignment will be described during the consent process. A certificate of confidentiality will be obtained. Procedures for youth consent and privacy for youth reports are not clearly described.

Data and Safety Monitoring Plan (Applicable for Clinical Trials Only):

Acceptable

- There will be an external data safety and monitoring board.

Inclusion of Women, Minorities and Children:

G1A - Both Genders, Acceptable

M1A - Minority and Non-minority, Acceptable

C1A - Children and Adults, Acceptable

- Parents and 11-14 year old children will be participants in the study. The sample is expected to be 78 percent White, 12 percent Latino, 5 percent Asian-American, and 5 percent African-American.

Vertebrate Animals:
Not Applicable (No Vertebrate Animals)

Biohazards:

Not Applicable (No Biohazards)

Resubmission:

- This is a revised application. The applicants have been extremely responsive to prior review. The clustering within schools will now be modeled in data analysis, the timeline is feasible, the procedures for youth reports are clearly described, teacher reports will be obtained and are described, the procedure for randomization is clear and appropriate, aim 3 that was considered to be premature has been dropped and aim 2 expanded. All of the changes have strengthened an application that was judged to be of high impact in prior review.

Budget and Period of Support:

Recommend as Requested

CRITIQUE 3:

Significance: 1
Investigator(s): 1
Innovation: 1
Approach: 1
Environment: 1

Overall Impact: This is an application for five years of funding to support the development and a study of the effectiveness of an online version of the Family Checkup (FCU), an in-school intervention program that has a proven record of success in reducing involvement in substance use and other

problem behaviors. An effective internet-accessible version of the Family Checkup would be a cost-effective, easily transportable intervention that could have an important impact on the behavioral health of children in middle school and beyond. The research team will include both of the developers of the Family Checkup intervention (Dr. Stormshak as principal investigator, and Dr. Thomas Dishion as consultant), as well as co-investigators (Drs. Seeley and Danaher) and consultants (Dr. Lochman) experienced in transforming in-school interventions into internet accessible interventions. The research environments of the University of Oregon and the Oregon Research Institute are excellent and should support the performance of the proposed research. The transformation of the established intervention to a web-based application is considered moderately innovative. Additional strengths of the application are the fact that parts of the online content have already been developed as part of an ARRA grant, the data analyses were appropriate, and there is ample documented school system support to generate subject flow for the proposed research. The investigators were highly responsive to the reviewer comments from the previous review, thereby eliminating the previous minor weaknesses, clarifying issues that were unclear, and strengthening the study. The proposed research should have considerable impact on the field and develop a useful, effective, transportable intervention for use nationally among middle school children.

Protections for Human Subjects:

Acceptable Risks and/or Adequate Protections

- The procedures to elicit informed consent and to protect the confidentiality of the data are satisfactory. A Certificate of Confidentiality from NIH will be applied for should the research be funded.

Data and Safety Monitoring Plan (Applicable for Clinical Trials Only):

Acceptable

- The DSMP is satisfactory.

Inclusion of Women, Minorities and Children:

G1A - Both Genders, Acceptable

M1A - Minority and Non-minority, Acceptable

C1A - Children and Adults, Acceptable

- Half of the children and 80 percent of the parents should be females. Approximately 78 percent of the sample will be European American, 12 percent Latino American, 5 percent Asian American, and 5 percent African American, consistent with the composition of the area in which the school districts are located.

Vertebrate Animals:

Not Applicable (No Vertebrate Animals)

Biohazards:

Not Applicable (No Biohazards)

Resubmission:

- The investigators were highly responsive to the previous reviews.

Budget and Period of Support:

Recommend as Requested

THE FOLLOWING RESUME SECTIONS WERE PREPARED BY THE SCIENTIFIC REVIEW OFFICER TO SUMMARIZE THE OUTCOME OF DISCUSSIONS OF THE REVIEW COMMITTEE ON THE FOLLOWING ISSUES:

PROTECTION OF HUMAN SUBJECTS (Resume): ACCEPTABLE

INCLUSION OF WOMEN PLAN (Resume): ACCEPTABLE

INCLUSION OF MINORITIES PLAN (Resume): ACCEPTABLE

INCLUSION OF CHILDREN PLAN (Resume): ACCEPTABLE

COMMITTEE BUDGET RECOMMENDATIONS: The budget was recommended as requested.

NIH has modified its policy regarding the receipt of resubmissions (amended applications). See Guide Notice NOT-OD-14-074 at http://grants.nih.gov/grants/guide/notice-files/NOT-OD-14-074.html. The impact/priority score is calculated after discussion of an application by averaging the overall scores (1-9) given by all voting reviewers on the committee and multiplying by 10. The criterion scores are submitted prior to the meeting by the individual reviewers assigned to an application, and are not discussed specifically at the review meeting or calculated into the overall impact score. Some applications also receive a percentile ranking. For details on the review process, see http://grants.nih.gov/grants/peer_review_process.htm#scoring.
